# Determinants of long acting reversible contraception utilization in Northwest Ethiopia: An institution-based case control study

**DOI:** 10.1371/journal.pone.0240816

**Published:** 2020-10-20

**Authors:** Kiros Terefe Gashaye, Adino Tesfahun Tsegaye, Solomon Mekonnen Abebe, Mulat Adefris Woldetsadik, Tadesse Awoke Ayele, Zelalem Mengistu Gashaw

**Affiliations:** 1 Department of Obstetrics and Gynecology, College of Medicine and Health Sciences, School of Medicine, University of Gondar, Gondar, Ethiopia; 2 Department of Epidemiology and Biostatistics, Institute of Public Health, College of Medicine and Health Sciences, University of Gondar, Gondar, Ethiopia; 3 Department of Nutrition, Institute of Public Health, College of Medicine and Health Sciences, University of Gondar, Gondar, Ethiopia; 4 Department of Obstetrics and Gynecology, College of Medicine and Health Sciences, School of Medicine, University of Gondar, Gondar, Ethiopia; 5 Department of Epidemiology and Biostatistics, Institute of Public Health, College of Medicine and Health Sciences, University of Gondar, Gondar, Ethiopia; 6 Department of Obstetrics and Gynecology, College of Medicine and Health Sciences, School of Medicine, University of Gondar, Gondar, Ethiopia; Queen’s University at Kingston, CANADA

## Abstract

**Background:**

Though long-acting reversible contraceptives (LARCs) are highly effective, have minimal side effects, require minimal follow-up, and are low cost, only 10% of contraceptives used in Ethiopia are LARCs. The reason for this low uptake is not understood at the country or regional level. Therefore, this study identified determinants of LARC utilization in Northwest Ethiopia.

**Methods:**

A facility-based unmatched case control study, using LARC users as cases and short- acting reversible contraception (SARC) users as controls, had been conducted at fourteen public health institutions in Northwest Ethiopia. A systematic random sampling technique was used to select participants with a 1:2 case to control ratio (n = 1167). Binary logistic regression analysis was used to identify determinants of LARC utilization among family planning service users.

**Results:**

Wealth status [AOR:1.87, 95%CI (1.08, 3.24)], history of abortion [AOR:2.69, 95%CI (1.41, 5.12)], limiting family size [AOR: 2.38, 95%CI (1.01, 5.62)], good knowledge about LARCs [AOR: 2.52, 95%CI (1.17, 5.41)], method convenience [AOR: 0.23, 95%CI (0.16, 0.34)], good availability of method [AOR:0.10 (0.05, 0.19)], less frequent visits to health facility [AOR:2.95, 95% CI(1.89, 4.62)], health care providers advice [AOR:10.69, 95%CI (3.27, 34.87)], partner approval [AOR:0.66, 95%CI (0.45, 0.97)], and favorable attitude towards LARCs [AOR:13.0, 95%CI (8.60, 19.72)] were significantly associated with LARC utilization.

**Conclusion:**

Professional support, favorable attitude towards LARC use, high economic status, history of abortion, advantage of less frequent visits, having good knowledge towards LARC and interest of limiting births were significantly associated with LARC Utilization. On the other hand, perceived method convenience, and contraception availability were inversely associated with it. Family planning education about the benefits of LARC should be done by health providers and media. Male involvement in the counselling and decision making about the advantage of using LARC may improve the negative influence of partners on LARC utilization. It is also recommended that, future qualitative research further explore perceptions of LARC use.

## Background

In today’s modern world, the voluntary control of fertility is especially important for couples to accomplish their individual goals and aspirations in addition to bearing children. The rapid growth of the human population in the world is another threat for survival for all, unless comparable economic growth of nations is attained. Hence birth control is of paramount importance for sustainable development and better living conditions [1–5].

Among modern birth control methods, long-acting reversible contraceptives (implants and Intra Uterine Contraceptive Device (IUCD)) are highly effective birth control technologies, determined to be one of the top tier methods by the World Health Organization [6]. Compared to short-acting reversible contraceptives (SARCs), long-acting reversible contraceptives (LARCs) have excellent effectiveness in avoiding unwanted pregnancy and its consequences [7–9]. They are also safe, with good continuation rates and low-cost. For these reasons, multiple studies have shown that they are the first-line contraceptive choices for all groups of clients including adolescents and women with chronic illness including HIV/AIDS [10–14]. Once placed properly, LARC users do not need to visit health care providers repeatedly, and LARCs are therefore considered to be “forgettable” for the women who use them. Studies also indicate that LARCs have failure rates of less than one percent [12], comparable to failure rates for tubal sterilization and vasectomy [15,16]. For this reason, LARCs are considered a means of chemical sterilization with an excellent potential to replace surgical sterilization techniques. Since LARCs are easily reversible, they are also good options to avoid post-sterilization regret [17]. In contrast to the very low failure rates of LARCs, the failure rate for SARC use among ideal users is 0.2% to 18% and the typical user failure rate is 6% to 28%, which may lead to higher rates of unwanted pregnancy and its consequences as compared to LARCs [11,18,19].

However, SARCs may be a better option for women who want to become pregnant sooner, and those who could benefit from the non-contraceptive benefits of SARCs such as decreasing incidence of ovarian and endometrial cancer and improvement of anemia [20].

Over all, there is ample evidence regarding the benefits of LARC use over SARC in terms of method effectiveness, side effects, safety and cost [18,21–23].

Use of such highly effective birth control methods is very important for communities suffering from high maternal and perinatal mortality and morbidity exacerbated by high fertility rates as well as large populations and unbalanced economic growth like Ethiopia [2]. In addition, LARCs have the potential to decrease the very high maternal and neonatal mortality of developing countries like Ethiopia [24].

Globally, LARC utilization among sexually active reproductive-age individuals is lower than SARC utilization [10,25]. Despite the overall increase in contraceptive prevalence in Ethiopia over the last two decades, the current proportion of SARC utilization over LARCs is 3.6-fold. In the Amhara region of Ethiopia, the modern contraception prevalence rate is 47%, and LARC use accounts for just 15% of this [26].

Reasons affecting LARC utilization are multidimensional including socio-demographic factors such as older age, higher level of education, better socioeconomic status and place of residence [27]. In addition, user related factors such as higher parity, history of unwanted pregnancy, history of smoking, misconception towards LARCs, lack of knowledge, negative attitude and fear of side effects affect LARC utilization [28,29]. Health facility related factors such as level of the health facility, availability of the method, partner influence are the other points affecting LARC use. Professional related factors such as level of training, competency of providers, and attitudes of providers may affect LARC utilization as well [30–32]. However, the reasons for low uptake of LARC are not well known in Ethiopia, particularly in our study site of Northwest Ethiopia. Therefore, the aim of this facility-based case control study was to identify the determinant factors affecting LARC utilization in Northwest Ethiopia, information which can then be used by health officials and policy makers seeking to increase LARC utilization in Ethiopia.

## Methods

### Study design and setting

A facility-based unmatched case control study was conducted after getting ethical approval from Institutional Review Board of the University of Gondar. The study aimed to assess the determinants of LARC utilization among modern family planning service users at health institutions in Northwest Ethiopia. The study was conducted by comparing women who use LARCs and SARCs among modern contraceptive users in Northwest Ethiopia’s public and non-governmental organizations (NGO) health facilities. Unlike descriptive cross-sectional studies, the case control design used in this study has the power to identify independent risk factors strongly associated with the specific outcome of interest; which, here was LARC use. This study was conducted from July 1st, 2016 to September 30th, 2016 in fourteen public and NGO health facilities located in Northwest Ethiopia. Northwest Ethiopia is part of the Amhara region, which includes Bahirdar, the current capital city of the Amhara region, and the city of Gondar. The topography of the area includes highlands and lowlands with different cultural, religious and ethnic groups. Currently, the study area has an estimated population of 9,011,940 of which approximately 50% are females [33].

Out of 41 health institutions (based on case load and geographic distribution) in the study area, we selected a total of 14 (34%) health facilities using simple random sampling method. The facilities included were five government hospitals (Dabark, Gondar, Metema, Debretabor and Felegehiwot); seven government health centers, (Debretabor, Kokit, Dabark, Gondar, Maraki, Bahirdar and Abaymado) and Gondar and Bahirdar Family Guidance Association (FGA) clinics.

The calculated sample size was proportionally allocated to each selected health facilities based on the previous consecutive three months average daily client flow of the units which was obtained from family planning registration log books. Every second person of the cases and controls were selected by using systematic random sampling procedure from family planning service users who were visiting the selected health institutions during the data collection period. The first client in each health facility was selected by lottery method. In all of the selected health facilities, family planning services are provided upon the request of clients. When clients in the family planning clinics seek contraceptives health care professionals provide them with the appropriate information about the available methods and offer them their method of choice.

### Participants and recruitment

#### Study population

The study population included all females using modern contraceptives who were attending family planning clinics in the selected fourteen health facilities during the study period.

#### Inclusion and exclusion criteria

Cases were all women using LARCs (IUCD, or implants) during the study period. Controls were women, using SARCs (oral contraceptive pills, condoms, or injectables) during the study period. Those who were seriously ill and/or unable to communicate for any reason were excluded.

#### Sample size calculation

The sample sizes for cases and controls were calculated based on the following assumptions: a 95% confidence level, power of 90%; and a 1:2 case to control. The required sample size was calculated using Epi Info version 7, and four variables from three previous studies conducted in similar settings were considered [34–36]. Variables such as residence, parity, working status, and number of children were taken from the three studies. Based on the calculation, the largest sample size out of the four variables was obtained from the variable “number of children,” and it was 1,061 [34]. With the consideration of a 10% non-response rate, the final sample size was determined to be 1,167 women.

### Study variables

The dependent variable was LARC utilization. The independent variables were socioeconomic and demographic factors (age, marital status, client’s level of education, partner’s educational status, occupation, residence, wealth index, religion, and ethnicity), user-related factors (fear of side effects, fear of needles/pain, knowledge, previous use of LARC, attitude towards LARC, method effectiveness, parity, history of unintended pregnancy, and smoking) and health facility related factors (type of health institution, location of health institution, availability of contraceptives (LARC or SARC). In addition, independent variables included partner influence (partner’s knowledge and attitude about contraceptives, women’s autonomy and decision making, lack of discussion with partners), and provider related factors (type of profession, experience and training was collected through document review; and, competency of method provision, provider beliefs and attitude, and provider counseling skills) were assessed using reported client perception. In addition, religion was included due to variation in guidance on family planning approaches across religions.

### Operational definitions

#### Long acting reversible contraceptive methods (LARCs)

Modern contraceptive methods are used for more than a year once the method is provided. In this study, intra-uterine contraceptive devices (IUCDs) and implants were the only LARC methods included.

#### Short acting reversible contraceptive methods (SARCs)

Modern contraceptive methods that are used for less than or equal to three months once the method is provided, such as oral contraceptive pills, injectables and condoms.

#### Knowledge of LARC

Women were considered to be knowledgeable if they were familiar with at least one of the LARCS (IUCD and/or implant).

Parity-women were classified as:

Nulliparous: women without a history of delivering a child
Primipara: women having delivered one child
Multiparous: women having delivered more than one child
Grand: multiparous- women delivered greater than or equal to five children

Gravidity: women were classified as:

Primigravida: women having a history of one pregnancy
Multi-gravida: women having more than one pregnancy

Abortion: loss of a fetus/unborn baby either intentionally or unintentionally before 28 weeks of gestation.

### Data collection procedures

The data was collected using a structured questionnaire in an exit interview of clients that came to the health facilities for family planning services. Participants provided written, informed consent before data was collected. The questionnaire was designed to obtain information on the socio-demographic characteristics of contraceptive users in public health facilities, as well as ascertain their reproductive history, utilization of modern contraceptive family planning use and factors affecting LARC methods. The questionnaire was prepared in English and translated into Amharic by a language professional. Seven interviewers and four supervisors were involved in the data collection process. The data collectors and supervisors took part in a two-day training covering topics including how to conduct an interview, rights of the study subjects, and ethical issues such as confidentiality. After the training, a pretest was administered to 60 women (5% of the sample size) in health facilities, which were not selected for this study. The questionnaire was modified after the pre-test was administered. On average, a single interview took around 30 minutes.

### Data processing and analysis

The data were entered into Epi info version 3.5.3, cleaned, and then transferred into STATA version 14.0 software for analysis. To capture wealth differences, Principal Component Analysis (PCA) scores were generated. The participants were asked about all possible household properties they owned. The common factor scores were summed up and ranked from lowest to highest.

Participants were said to be knowledgeable about LARC if they knew at least one of the LARCs. There were six Likert-scaled questions, which were used to assess the attitude of participants towards LARC. Those who scored above the mean (equivalent to 50 percent and above) were classified as having a favorable attitude and those who scored less than the mean (equivalent to less than 50 percent) were classified as having an unfavorable attitude. Binary logistic regression was used to identify socio-demographic determinants of LARC utilization. We incorporated variables, which had a p-value of less than 0.2 in the bivariable analysis in the final multivariable model. The Hosmer–Lemeshow goodness-of-fit test was used to test the overall goodness-of-fit. Adjusted odds ratios (AOR) with 95% confidence intervals (CI) were used to report the strength of the associations between LARC use and its explanatory variables.

## Ethical consideration

Ethical clearance was obtained from the University of Gondar Institutional Review Board. A formal letter was given to the regional health bureau, zonal and city administration health bureaus, hospitals, health centers, and FGA clinics. We obtained informed verbal consent from each woman enrolled in the study. The ethical review committees approved the procedure outlined in our study proposal. To maintain confidentiality, data containing personal identifiers of subjects were not collected and the data was kept locked.

## Results

### Socio-demographic characteristics of study participants

A total of 1,124 women of reproductive age were included in this study with a response rate of 96.3%. Of this, 382 [33.9%] were LARC users and 742 [66.0%] were SARC users. Seven hundred forty-nine [66.6%] participants were aged 20 to 29 years, and the overall mean [±SD] age of participants was 25.7 [± 5.6] years; 25.5 for SARC, and 26.0 years for LARC users, respectively.

Nine hundred twenty-eight [82.6%] participants were married and 963 (85.7%) were urban dwellers. Three hundred twenty-eight [29.2%] participants reported that they had never attended formal education. With regard to their economic status, 223 [19.8%] women were in the lowest/poorest quintiles and 226 [20.1%] were in the richest quintiles [Table 1].

**Table 1:** Socio-demographic characteristics of study participants in Amhara regional state, Northwest Ethiopia, 2016.

Variable	Category	SARC	LARC	Total
**Age in years**		**Frequency (%)**	**Frequency (%)**	**Frequency (%)**
	15–19	80(10.8)	36(9.4)	116 (10.3)
	20–24	256(34.5)	124(32.5)	380 (33.8)
	25–29	247(33.3)	122(31.9)	369 (32.8)
	30/34	84(11.3)	66(17.3)	150 (13.4)
	35–39	58(7.8)	24(6.3)	82 (7.3)
	≥40	17(2.3)	10(2.6)	27 (2.4)
**Residential location**				
	Urban	630(84.9)	333(87.2)	963 (85.7)
	Rural	112(15.1)	49(12.8)	161 (14.3)
**Marital status**				
	Married	604(81.4)	324(84.8)	928 (82.6)
	Single	94(12.7)	28(7.3)	122 (10.9)
	Divorced	24(3.2)	12(3.1)	36 (3.2)
	Widowed	10(1.3)	4(1.0)	14 (1.3)
	Separated	10(1.3)	14(3.7)	24 (2.1)
**Educational status**				
	Unable to read and write	141(19.00)	70(18.3)	211 (18.8)
	Able to read and write	62(8.4)	55(14.4)	117 (10.4)
	Primary education	143(19.3)	62(16.2)	205 (18.2)
	Secondary education	239(32.2)	92(24.1)	331 (29.5)
	Tertiary education	157(21.2)	103(26.9)	260 (23.1)
**Occupation**				
	Housewife	313(42.2)	158(41.4)	471 (41.9)
	Government employee	117(15.8)	64(16.7)	181 (16.1)
	Private employee	45(6.1)	39(10.2)	84 (7.5)
	Merchant	73(9.8)	47(12.3)	120 (10.7)
	Daily laborer	56(7.5)	21(5.5)	77 (6.8)
	Farmer	6(0.8)	10(2.6)	16 (1.4)
	Student	108(14.6)	30(7.8)	138 (12.3)
	Other	24(3.2)	13(3.4)	37 (3.3)
**Wealth status**				
	Poorest quintile	168(22.6)	55(14.4)	223 (19.8)
	Second quintile	166(22.4)	80(20.9)	246 (21.9)
	Third quintile	129(17.4)	78(20.4)	207 (18.4)
	Fourth quintile	135(18.2)	87(22.8)	222 (19.8)
	Richest quintile	144(19.4)	82(21.5)	226 (20.1)

### Reproductive characteristics of study participants

Two hundred ninety-two [29.1%] of the study participants had a history of early marriage [before age18]. Among the participants, 819 [72.9%], had a history of pregnancy. Among 776 women who had a history of parity, 410 [36.5%] were multi-parous and 310 [39.9%] Primipara. One hundred and forty-six participants [12.9%] had a history of abortion, and of these, 71, [6.3%] had a history of induced abortion. Fifty-five [4.9%] study subjects had a history of infantile death, and 28 [2.5%] clients had a history of neonatal death.

Of the 776 parous women, 126 [16.2%] had a history of home delivery in their last pregnancy. Among 817 women who had a history of pregnancy, 118 [14.4%] had a history of unwanted pregnancy, which was the result of no contraception utilization for 25 participants [21.2%], method forgotten for 45 participants [38.1%], rape for 20 participants [16.9%], or became pregnant while using contraceptive methods for 28 participants [23.7%] [Table 2].

**Table 2:** Reproductive characteristics of study participants in Amhara regional state, Northwest Ethiopia, 2016.

Variable	Category	Total n (%)	SARC Frequency (%)	LARC_ Frequency (%)
**Early marriage (N = 1,124)**				
	Yes	292 (29.1)	201(31.0)	91(25.7)
	No	710 (70.9)	447(69.0)	263(74.3)
**Gravidity (N = 1,124)**				
	None	305 (27.1)	225(30.3)	80(20.9)
	Primigravid	322 (28.6)	210(28.3)	112(29.3)
	2 to 4	410 (36.5)	249(33.6)	161(42.2)
	≥5	87 (7.7)	58(7.8)	29(7.6)
**Parity (N = 776)**				
	Primipara	310 (39.9)	204(41.2)	106(37.7)
	Multi-para	404 (52.1)	250(50.5)	154(54.8)
	Grand multi-para	62 (7.9)	41(8.3)	21(7.5)
**Abortion History (N = 1,124)**				
	Yes	146 (12.9)	669(90.2)	309(80.9)
	No	978 (87.0)	73(9.8)	73(19.1)
**History of infant death (N = 1,124)**				
	Yes	55 (4.9)	702(94.6)	367(96.1)
	No	1,069 (95.1)	40(5.4)	15(3.9)
**History of neonatal death (N = 1,124)**				
	Yes	28 (2.5)	724(97.6)	372(97.4)
	No	1096 (97.5)	18(2.4)	10(2.6)
**other’s age at first delivery (N = 1,124)**				
	<18	91 (11.7)	58(11.7)	33(11.7)
	18–24	548 (70.6)	354(71.5)	194(69.0)
	≥25	137 (17.6)	83(16.8)	54(19.2)
**Birth Interval (N = 466 (Multi-parous))**				
	<2 years	85 (18.2)	60(20.6)	25(14.3)
	2–5 years	329 (70.6)	194(66.7)	135(77.1)
	>5 years	52 (11.2)	37(12.7)	15(8.6)
**ANC for the last birth (N = 776 (parous))**				
	Yes	703 (90.6)	45(9.1)	28(10.0)
	No	73 (9.4)	450(90.9)	253(90.0)
**FP counseling during ANC (N = 703) |**				
	Yes	427 (60.7)	174(38.7)	102(40.3)
	No	276 (39.3)	276(61.3)	151(59.7)
**Place of last birth (N = 776 (parous))**				
	Health facility	650 (83.8)	416(84.0)	234(83.3)
	Home	126 (16.2)	79(16.0)	47(16.7)
**Wanted status of last pregnancy (N = 817)**				
	Unwanted	118 (14.4)	64(12.4)	54(17.9)
	Wanted	699 (85.6)	451(87.6)	248(82.1)
**PNC for the last birth (N = 776 (parous))**				
	No	320 (41.2)	212(42.8)	108(38.4)
	Yes	456 (58.8)	283(57.2)	173(61.6)
**FP counseling during PNC (N = 456) |**				
	No	75 (16.4)	47(16.6)	28(16.2)
	Yes	381 (83.5)	236(83.4)	145(83.8)
**Breast feeding for the most recent birth (N = 776)**				
	No	41 (5.3)	26(5.2)	15(5.3)
	Yes	735 (94.7)	469(94.8)	266(94.7)
**Breast feeding duration (N = 735)**				
	<6 months	148 (20.1)	90(19.2)	58(21.8)
	6 months to 2 years	433 (58.9)	274(58.4)	159(59.8)
	> 2 years	154 (20.9)	105(22.4)	49(18.4)
**Reproductive intention (N = 1124)**				
	Want to space	541 (48.1)	336(45.3)	205(53.7)
	Want to limit	127 (11.3)	78(10.5)	49(12.8)
	Undecided	351 (31.2)	248(33.4)	103(27.0)
	Want to have a child soon	105 (9.3)	80(10.8)	25(6.5)

### Knowledge, attitude and utilization of contraceptive methods

**Fig 1:**
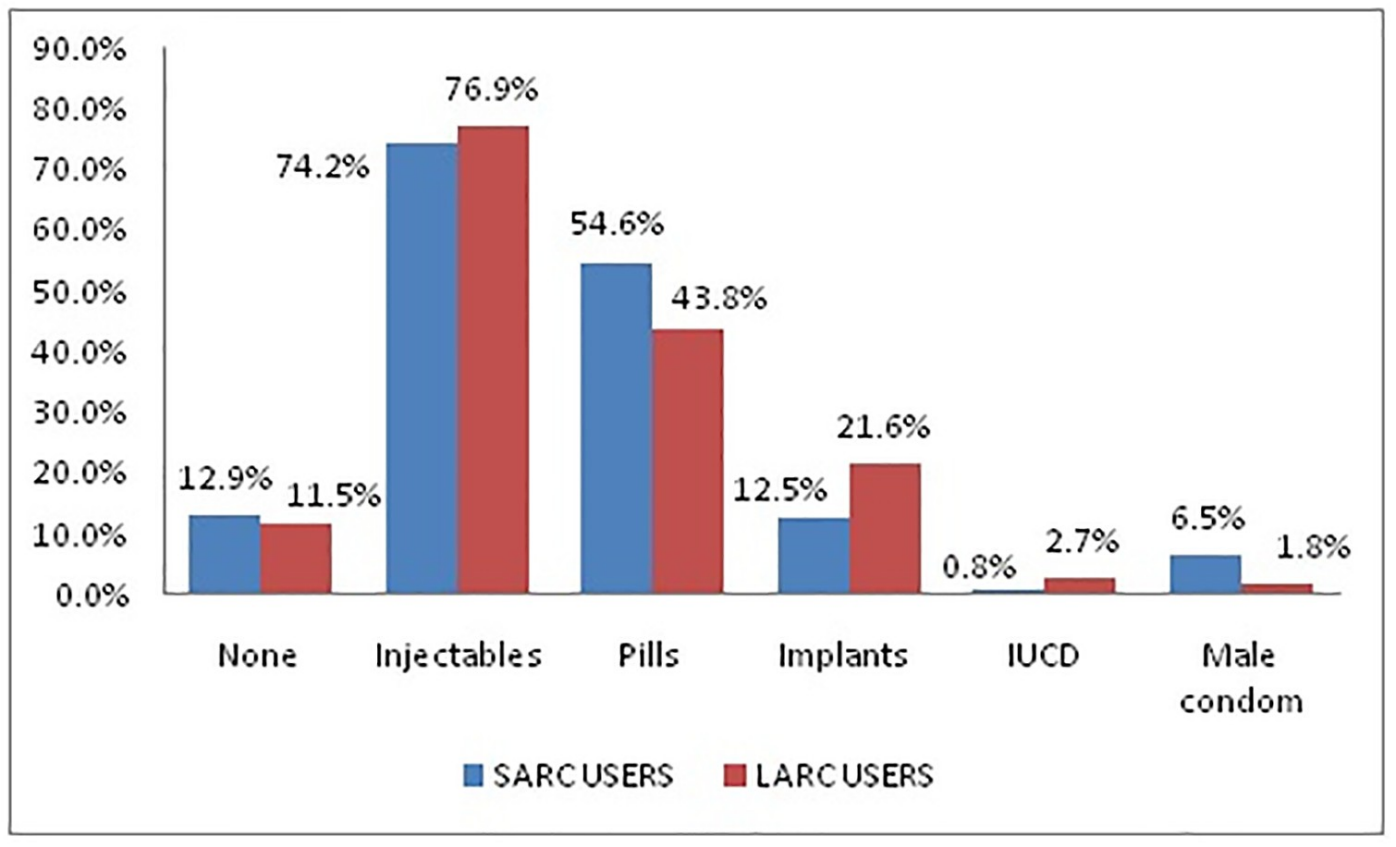
History of previous contraceptive methods used by SARC and LARC user women (N = 1124) in Amhara regional state, Northwest Ethiopia, 2016.

Only 86 [7.6%] of all participants had never heard about LARCs while 991[88.2%] study participants heard some information about implants, and 731 [65.0%] about IUCDs. Regarding their knowledge about LARCs, 1,025 [91.2%] of the participants were knowledgeable. Six hundred three [53.6%] clients had favorable attitude towards LARC utilization. Nine hundred eighty-four [87.5%] clients had a previous history of contraceptive utilization, with injectables being the top to be used by 739 [75.1%] clients (Fig 1).

With respect to side effects of the methods used previously, 228 [23.2%] clients reported irregular vaginal bleeding, 145 [14.7%] weight gain, 130 [13.2%] nausea and vomiting, 70 [7.1%] abdominal pain and 19 [1.9%] insertion site infection.

### Current contraceptive methods

**Fig 2:**
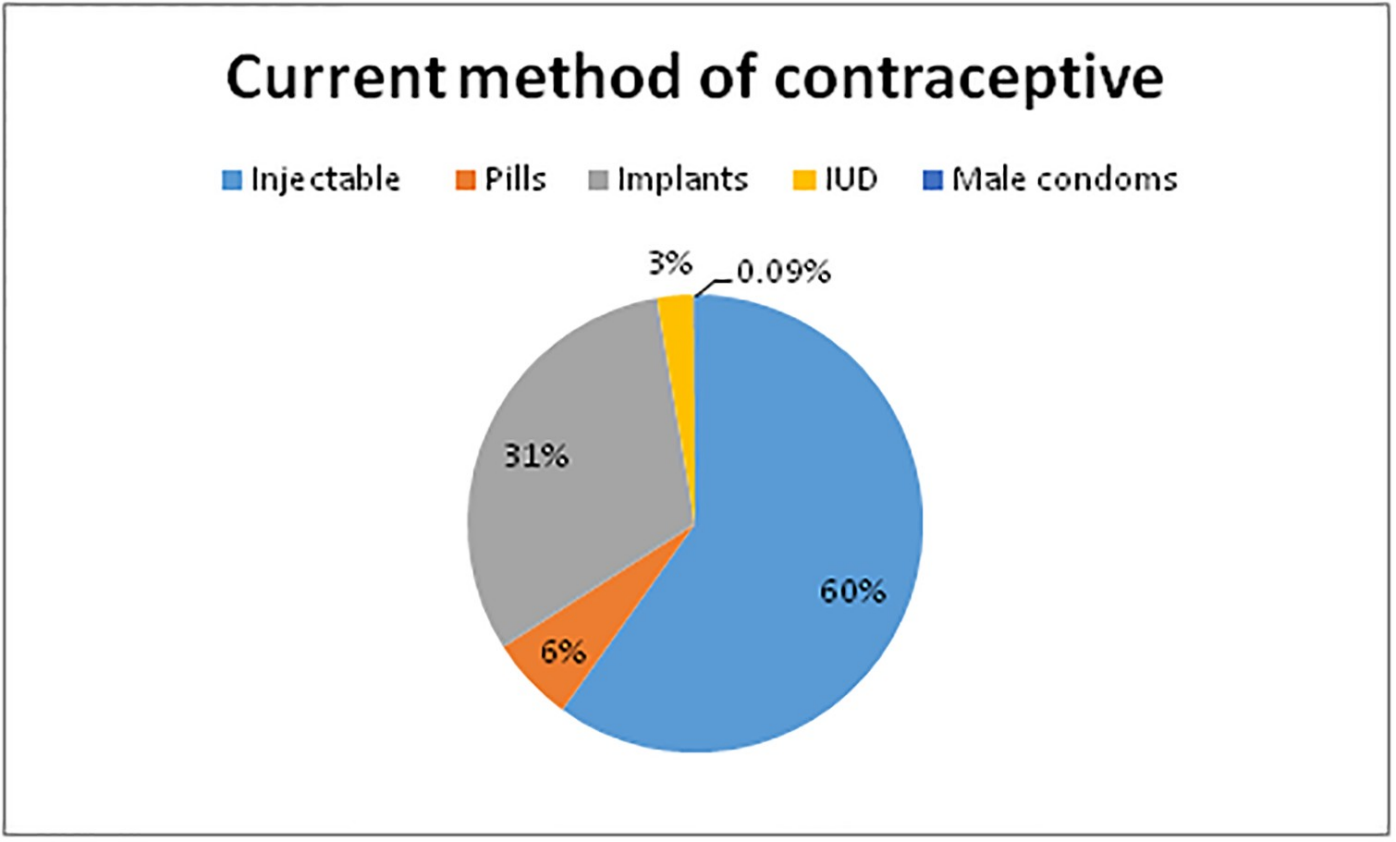
Current contraceptive methods used by women in the Amhara regional state, Northwest Ethiopia, 2016.

As shown in Fig 2, 382 [34.0%] were LARC users [implant and IUCD] and 742 [66.0%] were SARC users [injectables, pills, and/or male condoms].

Nurses were the leading providers of contraception, providing it for 482 [42.9%] clients. About 983 [87.5%] of the clients reported that, the information given during family planning counseling was important, and 957 [85.9%] of the clients said that they also trust the information given.

When study participants currently using a LARC were asked why they chose LARCs, instead of SARCs, 234 [61.3%] reported that they did not desire to have a pregnancy soon and 228 [59.69%] reported that LARCs had less side effects than SARCs [Table 3].

**Table 3:** Reasons for using LARC among women who were using modern contraceptive methods in Amhara regional state, Northwest Ethiopia, 2016.

WHY LARC (N = 382)		
		Frequency (%)
Fewer side effects than SARCs	Yes	228 (59.7)
Highly effective	Yes	90 (23.6)
No effect on fertility	Yes	125 (32.7)
Desire not to have pregnancy soon	Yes	234 (61.3)
Religious permission	Yes	1 (0.3)
Medical problem*	Yes	26 (6.8)
Easily accessible	Yes	10 (2.6)
Acceptable in my culture	0/No one say yes	
Health Professionals’ advise	Yes	43 (11.3)
Important partner/others influence	Yes	3 (0.8)
Convenient to me	Yes	200 (52.3)
No other choice	Yes	5 (1.3)

*HIV, Diabetes Mellitus, Hypertension, Renal Failure, Congestive Heart Failure.

Controls were also asked the reason for not utilizing LARCs, and 441 [59.43%] reported that this was due to fear of side effects and 116 [15.63%] indicated a desire to become pregnant soon [Table 4].

**Table 4:** Reasons for not using LARC among women who use modern contraceptive methods in Amhara regional state, Northwest Ethiopia, 2016.

WHY NOT LARC		
		Frequency (%)
Fear of side effects	No	301 (40.6)
	Yes	441 (59.4)
Less effective		
	No	718 (96.8)
	Yes	24 (3.2)
Fear of infertility		
	No	551 (74.3)
	Yes	191 (25.7)
Desire to become pregnant soon		
	No	626 (84.4)
	Yes	116 (15.6)
Religious prohibition		
	No	734 (98.9)
	Yes	8 (1.1)
Medical problem		
	No	726 (97.8)
	Yes	16 (2.2)
Lack of commodity/absence of method		
	No	663 (89.4)
	Yes	79 (10.6)
Rumors/complains from other users		
	No	533 (71.8)
	Yes	209 (28.2)
Unacceptable in my culture		
	No	726 (97.8)
	Yes	16 (2.2)
The advice of others was important		
	No	645 (86.9)
	Yes	97 (13.1)
Lack of knowledge		
	No	589 (79.4)
	Yes	153 (20.6)
Fear of needle and pain		
	No	609 (82.1)
	Yes	133 (17.9)
Inconvenient for me		
	No	593 (79.9)
	Yes	149 (20.1)

### Method choice and decision making

Among the respondents, 1,085 [96.53%] reported using their most preferred method of contraception. Of those using contraception, 830 [73.84%] clients said they had discussed the choice of the current method with their partner, and 632 [56.23%] clients needed their husband/partner’s approval to choose the method. Regarding their decision to use their current contraceptive method, 569 [51.08%] chose by themselves and 502 [45.06%] made the decision jointly with their partners.

### Factors associated with LARC utilization

All variables with a p-value of less than or equal to 0.2 in the bivariable model was fitted to the multivariable model. Variables included in the multivariable model were wealth status, history of abortion, desire to limit child bearing, knowledge about LARC, convenience of the method of choice, perceived ease of reversibility of the method, easy availability of the method of choice, less frequent visits, advise by health professionals, husband/partner approval, and favorable attitude towards LARC, as each was significantly associated with LARC utilization [Table 5].

**Table 5:** Factors associated with LARC utilization among women who were using modern contraceptive methods in Amhara regional state, Northwest Ethiopia, 2016.

Variable	Category	SARC User n(%)	LARC User n(%)	COR (95% CI)	AOR (95% CI)
Age in years					
	15–19	80(10.78)	36(9.42)	1	1
	20–24	256(34.5)	124(32.46)	1.08 (0.69, 1.68)	0.88(0.48, 1.64)
	25–29	247(33.29)	122(31.94)	1.10(0.7, 1.72)	0.78(0.38, 1.59)
	30/34	84(11.32)	66(17.28)	1.75 (1.05, 2.90)	0.70(0.29, 1.68)
	35–39	58(7.82)	24(6.28)	0.92 (0.50, 1.70)	0.54(0.18, 1.56)
	≥40	17(2.29)	10(2.62)	1.31 (0.55, 3.13)	0.68(0.16, 2.88)
**Wealth status**					
	Poorest quintile	168(22.64)	55(14.40)	1	1
	Second quintile	166(22.37)	80(20.94)	1.47(0.98, 2.21)	1.57(0.91, 2.69)
	Third quintile	129(17.39)	78(20.42)	1.85(1.22, 2.79)	1.37(0.78, 2.34)
	Fourth quintile	135(18.19)	87(22.77)	1.97(1.31, 2.96)	1.87(1.08, 3.24)*
	Richest quintile	144(19.41)	82(21.47)	1.74(1.16, 2.61)	1.69(0.96, 2.98)
**Abortion History**					
	No	669(90.16)	309(80.89)	1	1
	Yes	73(9.84)	73(19.11)	2.16(1.52, 3.07)	2.69(1.41, 5.12)*
**Reproductive intention**					
	Want to space out their births	336(45.28)	205(53.66)	1.95(1.21, 3.16)	1.82(0.96, 3.44)
	Want to limit their number of births	78(10.51)	49(12.83)	2.01(1.13, 3.57)	2.38(1.01, 5.62)*
	Undecided	248(33.42)	103(26.96)	1.33(0.80, 2.20)	1.53(0.75, 3.09)
	Want to have a child soon	80(10.78)	25(6.54)	1	1
**LARC knowledge**					
	Not Knowledgeable	79(10.65)	20(5.24)	1	1
	Knowledgeable	663(89.35)	362(94.76)	2.16(1.29, 3.58)	2.52(1.17, 5.41)*
**Perceived convenience of the method of choice**					
	No	181(24.39)	198(51.83)	1	1
	Yes	561(75.61)	184(48.17)	0.29(0.23, 0.39)	0.23(0.16, 0.34)*
**Perceived ease of reversibility of the method of choice**					
	No	533(71.83)	254(66.49)	1	1
	Yes	209(28.17)	128(33.51)	1.28(0.98, 1.67)	1.47(1.00, 2.17)
**Easy of availability of the method of choice**					
	No	566(76.28)	368(96.34)	1	1
	Yes	176(23.72)	14(3.66)	0.12(0.07, 0.21)	0.10(0.05, 0.19)*
**Less frequent follow-up visits required**					
	No	660(88.95)	270(70.68)	1	1
	Yes	82(11.05)	112(29.32)	3.34(2.43, 4.59)	2.95(1.89, 4.62)*
**Who decided to use the current method**					
	Self	390(53.13)	179(47.11)	1	1
	Mainly husband/partner	8(1.09)	4(1.05)	1.09(0.32, 3.66)	2.00(0.32, 12.34)
	Joint decision	330(44.96)	172(45.26)	1.14(0.88, 1.47)	1.23(0.85, 1.79)
	Health care provider advise	6(0.82)	25(6.58)	9.08(3.66, 22.52)	10.69(3.27, 34.87)*
**Husband/partner approval**					
	No	302(40.7)	137(35.86)	1	1
	Yes	440(59.30)	245(64.14)	1.23(0.95, 1.58)	0.66(0.45, 0.97)*
**Attitude toward LARC**					
	Unfavorable attitude	472(63.61)	49(12.83)	1	1
	Favorable attitude	270(36.39)	333(87.17)	11.88(8.49, 16.61)	13.02(8.60, 19.72)*

Note

*P-value < 0.05.

The odds of using LARC were 1.9 times higher [AOR: 1.9, 95% CI (1.1, 3.2)] among women who were in the fourth quintile wealth status than those in the poorest quintile. Women who had a history of abortion were 2.7 times more likely to use LARC than those who had never had an abortion [AOR: 2.7, 95% CI (1.4, 5.1)]. Women who reported wanting to limit their births were 2.4 times more likely to use LARCs than those who reported wanting to have a child soon [AOR: 2.4, 95% CI (1.01, 5.6)]. Women who had good knowledge about LARCs were 2.5 times more likely to use LARC than those who had poor knowledge [AOR: 2.5, 95% CI (1.2, 5.4)].

Women who chose LARC were 13 times more likely to have favorable attitude towards LARCs than SARC users [AOR: 13.0, 95% CI (8.6, 19.7)] and LARC users were more than 10 times more likely to choose their method due to the advice of health care professionals than SARC users [AOR: 10.7, 95% CI (3.3, 34.9)]. In addition, LARC users were 1.5 times more likely to choose their method due to its ease of reversibility than SARC users [AOR: 1.5, 95% CI (1.0, 2.2)] and it’s also 3.0 times more likely to choose their method due to less frequent visits than SARC users [AOR: 3.0, 95% CI (1.9, 4.6)]. On the other hand LARC users were 90% less likely to choose their method due to its ease of availability than SARC users [AOR: 0.10 (0.05, 0.19)] and it’s also 77% less likely to choose their method due to its convenience than SARC users [AOR: 0.23, 95% CI (0.16, 0.34)]. Approval of their partner/husband to choose their method need were 34% less likely to occur among LARC than SARC users the [AOR: 0.66, 95% CI (0.45, 0.97)].

## Discussion

The purpose of this study was to identify determinants of LARC utilization among women accessing facility-based medical care at public health institutions in Northwest Ethiopia. In this study, higher economic status, history of spontaneous or induced abortion, desire to limit family size, good knowledge of LARCs, less frequent medical visits, advise of health professionals, and a positive attitude of clients toward LARCs were associated with LARC utilization. In contrast, perceived method convenience, easy availability of the method and partner influence, were negatively associated with using LARC.

In this study, clients with the highest income level were found to use LARCs more often than those who earn the least amount of income. This finding is consistent with studies conducted in West Iran, Zambia and Ethiopia in which women who utilize LARC were wealthier than those who did not use LARC [36,37]. In the context of Ethiopia, many wealthy people are relatively educated, live in urban areas, and have easier access to media and information, which could lead to well-informed understanding of the available methods and thus, the use of LARCs. In contrast, a systematic review conducted in France showed that women in difficult financial situations were positive towards IUCD use [31]. One qualitative study exploring obstacles to the use of LARCs among women in Seattle, USA, revealed that expense and billing were major barriers [38]. Taken together, these studies support the notion that both wealthy and poor women in the developed and developing world use LARCs. Differences in patterns of LARC use among these studies can be explained by differences in the study population and settings. From this point, it would be of paramount importance for policy makers and health planners to be focused on strategies that are more feasible and faster to provide LARCs at a subsidized rate while working to empower women on economic activities.

In this study, we found that participants who are currently using modern contraceptives and have history of abortion were 2.69 times more likely to use LARCs than their counterparts who did not have a history of abortion. This finding is consistent with studies set in France and Ethiopia (Adigrat town) in which history of unintended pregnancy was positively associated with LARC utilization [31,39,40]. Patients who had history of abortion may be highly motivated to utilize a very effective method of contraception. This was also evidenced in a study conducted in the United States in which women who were offered immediate post-abortion contraception were more likely to choose an IUCD or an implant than women without a history of a recent abortion [41]. It would be good for health managers to work in creation of public awareness about the effectiveness of LARCs in preventing unintended pregnancy.

In this study, women who wanted to limit their birth were 2.38 times more likely to use LARC than those who wanted to have a child more quickly. This finding is consistent with the results of a study that took place in 14 European countries, France and Ethiopia in which LARCs were mainly used by women who had children and did not wish to have any more [30,31,42]. One of the most important benefits of LARCs is their long duration of use once they are placed. For this reason, more women who want to limit their family size may tend to utilize LARCs which are long term, effective and reversible. As such, LARCs may be used as a replacement for surgical sterilization, and because of their ease of reversibility, they may help women to avoid post-sterilization regret.

In the present analysis, lack of knowledge and information about long-acting family planning was found to affect LARC use. Women who had good knowledge about LARCs preferred to use LARCs. This is similar to studies done elsewhere which showed that better/good knowledge led clients to LARCs utilization while lack of knowledge about LARCs was negatively associated with their use [35,40,43–53].

Several cross-sectional studies conducted in Europe and Africa, as well as a qualitative study in Australia found a similarly negative effect of lack of knowledge on LARC utilization [40,43,46,54,55]. LARCs are highly effective in preventing unwanted pregnancy with great convenience, fewer side effects and less cost than SARCs [22,23,56]. Clients with good knowledge will understand that LARCs are highly effective and safe choices, and therefore utilize them. Activities aimed at increasing the public’s knowledge about the benefits of LARCs should be a focus of the health sector.

In this study, the family planning provider’s advice on decision-making of clients is found to significantly impact the utilization of LARCs. Clients advised by health care professionals were more likely to use LARCs than those who chose by themselves. This is consistent with a study done in France which showed that professional training and experience was significantly associated with LARC use irrespective of specialty [31,39,57]. Another study conducted in West Ethiopia found that, discussions with health care providers about long acting and permanent contraceptive methods positively affected LARC utilization [42,58].

Moreover, a qualitative study in Australia showed that providers’ lack of confidence and support for LARC insertion had a negative effect on LARC use [46]. Providers have a responsibility to clearly communicate and support their clients to choose the method which best fits their personal circumstance. It would be good to provide in-service training for providers on how to support their clients in explaining the effectiveness of LARCs during their counselling session.

In the present study, perceived convenience of the method was less likely to be reported among LARC users, a finding that is counter to two studies demonstrating the importance of convenience in the selection of LARCs as a contraceptive method [30,59]. This discrepancy may be due to rumors, myth and misconceptions, as seen in El Salvador negatively affecting IUCD utilization [60], and fear of procedure related pain and side effects [61] may additionally account for this discrepancy. Advocating for the convenience of LARCs and dispelling myths, misconceptions and rumors should be a strategic approach for increasing LARC uptake.

In this study, clients who were using LARCs didn’t choose it due to its easy availability, which is contrary to Rwanda where availability of LARCs was seen to increase implant utilization in postpartum HIV-positive women [62]. Similarly, the issue of access was one of the limiting factors for LARC utilization in Australia [46]. This may be due to, LARCs services being free and easily accessed in Ethiopia, and therefore the issue of availability may not be a concern for clients. The second reason may be lack of awareness. Due to earlier introduction of SARCs, which are more well-known contraceptive methods within the community studied here, clients prefer to use SARCs and may not bother to inquire about the availability of LARCs [26].

Moreover, the influence of the husband/partner also played a role in decision-making which negatively affects LARC utilization in this study. This finding is consistent with studies conducted in the Tigray and Oromia regions of Ethiopia where no or limited support from male partners and lack of discussion between partners was an obstacle to LARC use [34,63]. In another study in the Tigray region of Ethiopia, women who selected their contraceptive method alone were more likely to use LARCs as compared to those who decided jointly with their partners [40]. Similarly, married women with partners who did not permit them to use LARCs, partner’s poor knowledge of LARCs, and negative attitudes about LARCs were negatively associated with LARC use. However, this is in contrast to studies done in Uganda, Zambia, West Ethiopia and a case-control study from the Ethiopian EDHS 2011, in which joint decision making, women who live with their partners, and women’s attitude that male partners’ choice influences their contraceptive decisions were positively associated with current use of LARCs [34,36,37,44,58,63,64]. Some male partners may not understand the benefits of LARCs, and some may even have misconceptions that do not support their utilization.

In this study, women who had a positive attitude towards LARCs were more likely to use LARC than those who did not. This study is consistent with studies completed in the Wolayta zone, the town of Adigrat and the West Arsi zone of Ethiopia which found that women with positive attitudes towards LARCs were positively influenced to use them [40,55,65].

### Strength and limitation of the study

This study tried to incorporate as many determinants of LARC utilization as possible. In the present study, selection bias has been minimized because both study cases and controls were recruited from the same health facilities using systematic random sampling.

Temporal relationships can be established since the study used incident cases, and recall bias was not a major problem since the majority of questions inquired about basic healthcare information and personal opinions. However, it is possible that recall bias affected some variables such as age at marriage, age at first birth, history of ANC and PNC. The findings of this study may not be generalizable to community members who do not attend health facilities.

### Conclusion and recommendations

Professional support, favorable attitude towards LARC use, high economic status, history of abortion, advantage of less frequent visits, having good knowledge towards LARC and interest of limiting births were significantly associated with LARC Utilization. Whereas perceived method convenience, and contraception availability were inversely associated with it. Family planning education about the benefits of LARC should be done by health providers and media. Male involvement in the counselling and decision making about the advantage of using LARC may improve the negative influence of partners on LARC utilization. It is also recommended that, future researchers can try to explore the deep perception of LARC users using qualitative method.

## Supporting information

S1 Tool Tool used for data collection. (ZIP)

S1 Data Data underlying the study findings. (DTA)

## References

[1] S.Berek, J., Berek and Novak’s Gynecology. 2012.

[2] Tsegaye Tegenu, P.D., Exponential Population Growth and Carrying Capacity of the Ethiopian Economy. July 7, 2013.

[3] bongaarts, j., et al., Family Planning Programs for the 21st Century Rationale and design. 2012.

[4] World Health Organization Department of Reproductive Health and Research, Johns Hopkins Bloomberg School of Public Health Center for Communication Programs Knowledge for Health Project, and United States Agency for International Development (USAID) Bureau for Global Health Office of Population and Reproductive Health, Family Planning A GLOBAL HANDBOOK FOR PROVIDERS Evidence-based guidance developed through worldwide collaboration. 2011.

[5] Bailey M.J., Malkova O., and Norling J., Do Family Planning Programs Decrease Poverty? Evidence from Public Census Data. CESifo Econ Stud, 2014 60(2): p. 312–337. 10.1093/cesifo/ifu011 25346655 PMC4206087

[6] WHO, Family Planning A GLOBAL HANDBOOK FOR PROVIDERS. 2011.

[7] Hubachera D., Mavranezoulib I., and McGinna E., Unintended pregnancy in sub-Saharan Africa: magnitude of the problem and potential role of contraceptive implants to alleviate it. Contraception, 2008 78(1): p. 73–78. 10.1016/j.contraception.2008.03.002 18555821

[8] Hubacher D., Mavranezouli I., and McGinn E., Unintended pregnancy in sub-Saharan Africa: magnitude of the problem and potential role of contraceptive implants to alleviate it. Contraception. 78(1): p. 73–78. 10.1016/j.contraception.2008.03.002 18555821

[9] Hubacher D., et al, Short-acting and long-acting reversible contraceptive methods: comparing effectiveness using a partially randomized patient preference trial. Contraception, 2015 92(4): p. 382. 10.1016/j.contraception.2014.11.006 PMC4370363 25500324

[10] Joshi R., Khadilkar S., and Patel M., Global trends in use of long-acting reversible and permanent methods of contraception: Seeking a balance. Int J Gynaecol Obstet, 2015 131(1): p. 60–3. 10.1016/j.ijgo.2015.04.024 26433510

[11] Hoffman, B.L., et al., Williams_Gynecology_3rd_Edition. 2016.

[12] Blumenthal P.D., Voedisch A., and Gemzell-Danielsson K., Strategies to prevent unintended pregnancy: increasing use of longacting reversible contraception. Human Reproduction Update, 2011 17(1): p. 121–137. 10.1093/humupd/dmq026 20634208

[13] Mestad R., et al, Acceptance of long-acting reversible contraceptive methods by adolescent participants in the Contraceptive CHOICE Project. Contraception, 2011 84(2011): p. 493–498. 22018123 10.1016/j.contraception.2011.03.001 PMC3505875

[14] Black I., K., et al, Why do women experience untimed pregnancies? A review of contraceptive failure rates. Best Practice & Research Clinical Obstetrics and Gynaecology, 2010 24(4): p. 443–455. 20335073 10.1016/j.bpobgyn.2010.02.002

[15] Schwing P.J. and AGuess H., Safety and effectiveness of vasectomy. Fertility and Sterility, 2000 73(5): p. 923–936. 10.1016/s0015-0282(00)00482-9 10785217

[16] Aileen M.Gariepy M.D.C., smith Kenneth J., Xiao Xu, Probability of pregnancy after sterilization: a comparison of hysteroscopic versus laparoscopic sterilization. contraception, 2014 90(2): p. 174–181. 10.1016/j.contraception.2014.03.010 24767963

[17] Kumari S.S., Permanent Sterilisation to Long-Acting Reversible Contraception: Is a Paradigm Shift Necessary? J Obstet Gynaecol India, 2016 66(3): p. 149–53. 10.1007/s13224-016-0866-2 27298522 PMC4870672

[18] Stoddard A., McNicholas C., and Peipert J.F., Efficacy and Safety of Long-Acting Reversible Contraception. Drugs 2011 71(8): p. 969–980. 10.2165/11591290-000000000-00000 21668037 PMC3662967

[19] Xu H., et al, Contraceptive Failure Rates of Etonogestrel Subdermal Implants in Overweight and Obese Women. Obstet Gynecol., 2012 120(1): p. 21–26. 10.1097/AOG.0b013e318259565a 22678035 PMC4043143

[20] Carey M.S. and Allen R.H., Non-contraceptive uses and benefits of combined oral contraception. The Obstetrician and Gynecologist, 2012 14: p. 223–8.

[21] Winner B., et al, Effectiveness of Long-Acting Reversible Contraception. Obstetrical & Gynecological Survey, 2012 67(9): p. 552–553.

[22] Winner B., et al, Effectiveness of Long-Acting Reversible Contraception. The new england journal of medicine, 2012 366(21): p. 1998–2007. 10.1056/NEJMoa1110855 22621627

[23] Mavranezouli I., The cost-effectiveness of long-acting reversible contraceptive methods in the UK: analysis based on a decision-analytic model developed for a National Institute for Health and Clinical Excellence (NICE) clinical practice guideline. Human Reproduction, 2008 23 (6): p. 1338–1345. 10.1093/humrep/den091 18372257

[24] Ahmed S., et al, Maternal deaths averted by contraceptive use: an analysis of 172 countries. The Lancet, 2012 380(9837): p. 111–125. 10.1016/S0140-6736(12)60478-4 22784531

[25] Blumenthal P.D., Voedisch A., and Gemzell-Danielsson K., Strategies to prevent unintended pregnancy: increasing use of long-acting reversible contraception. Hum Reprod Update, 2011 17(1): p. 121–37. 10.1093/humupd/dmq026 20634208

[26] Central Statistical Agency, Ethiopian demographic and health survey. 2016.

[27] Haimovich S., Profile of long-acting reversible contraception users in Europe. Eur J Contracept Reprod Health Care, 2009 14(3): p. 187–95. 10.1080/13625180902741436 19565416

[28] Melka A.S., Tekelab T., and Wirtu D., Determinants of long acting and permanent contraceptive methods utilization among married women of reproductive age groups in western Ethiopia: a cross-sectional study. Pan Afr Med J, 2015 21: p. 246 10.11604/pamj.2015.21.246.5835 26523185 PMC4607985

[29] Habtamu A., et al, Determinants of long-acting contraceptive utilization among married women of reproductive age in Aneded district, Ethiopia: a case-control study. BMC Res Notes, 2019 12(1): p. 433 10.1186/s13104-019-4445-3 31319878 PMC6637530

[30] Haimovich S., Profile of long-acting reversible contraception users in Europe. The European Journal of Contraception & Reproductive Health Care, 2009 14(3): p. 187–195. 19565416 10.1080/13625180902741436

[31] Moreau C., et al, Trends and determinants of use of long-acting reversible contraception use among young women in France: results from three national surveys conducted between 2000 and 2010. Fertil Steril, 2013 100(2): p. 451–8. 10.1016/j.fertnstert.2013.04.002 23663994

[32] Mekonnen W. and Worku A., Determinants of low family planning use and high unmet need in Butajira District, South Central Ethiopia. Reprod Health, 2011 8(37): p. 1–8. 22151888 10.1186/1742-4755-8-37 PMC3248357

[33] Central statstical agency of Ethiopia, POPULATION and HOUSING CENSUS OF ETHIOPIA. 2007.

[34] Alemayehu M., et al, Rural women are more likely to use long acting contraceptive in Tigray region, Northern Ethiopia: a comparative community-based cross sectional study. BMC Womens Health, 2015 15(71): p. 1–8. 26341405 10.1186/s12905-015-0229-7 PMC4560916

[35] Alemayehu M., Belachew T., and Tilahun T., Factors associated with utilization of long acting and permanent contraceptive methods among married women of reproductive age in Mekelle town, Tigray region, north Ethiopia. BMC Pregnancy Childbirth, 2012 12(6): p. 1–9. 22280163 10.1186/1471-2393-12-6 PMC3297532

[36] Teferra A.S. and Wondifraw A.A., Determinants of long acting contraceptive use among reproductive age women in Ethiopia: Evidence from EDHS 2011. Science Journal of Public Health, 2015 3(1): p. 143–149.

[37] Mutombo N. and Bakibinga P., The effect of joint contraceptive decisions on the use of Injectables, Long-Acting and Permanent Methods (ILAPMs) among married female (15–49) contraceptive users in Zambia: a cross- ectional study. reproductive health, 2014 11(51): p. 1–8. 24993034 10.1186/1742-4755-11-51 PMC4098924

[38] Gilmore K., et al, Providing long-acting reversible contraception services in Seattle school-based health centers: key themes for facilitating implementation. J Adolesc Health, 2015 56(6): p. 658–65. 10.1016/j.jadohealth.2015.02.016 26003582

[39] Moreau C., et al, IUD use in France: women’s and physician’s perspectives. Contraception, 2014 89(1): p. 9–16. 10.1016/j.contraception.2013.10.003 24239330

[40] Gebreyesus B., Berhe S., and Bayray A., ASSESSMENT OF LONG ACTING AND PERMANENT CONTRACEPTIVE METHOD UTILIZATION AND ASSOCIATED FACTORS AMONG MARRIED WOMEN OF REPRODUCTIVE AGE GROUP IN ADIGRAT TOWN, TIGRAY REGION, ETHIOPIA. AMERICAN JOURNAL OF ADVANCES IN NURSING RESEARCH, 2015 2(1): p. 36–45.

[41] Madden T., et al, Comparison of contraceptive method chosen by women with and without a recent history of induced abortion. Contraception, 2011 84(6): p. 571–7. 10.1016/j.contraception.2011.03.018 22078185 PMC3563318

[42] Teferra A.S. and Wondifraw A.A., Determinants of Long Acting Contraceptive Use among Reproductive Age Women in Ethiopia: Evidence from EDHS 2011. Science Journal of Public Health, 2015 3(1): p. 143–149.

[43] Bracken J.G., A., Young women’s attitudes towards, and experiences of, long-acting reversible contraceptives. Eur J Contracept Reprod Health Care, 2014 19(4): p. 276–84. 10.3109/13625187.2014.917623 24882426

[44] Anguzu R., et al, Knowledge and attitudes towards use of long acting reversible contraceptives among women of reproductive age in Lubaga division, Kampala district, Uganda. BMC Res Notes, 2014 7: p. 153 10.1186/1756-0500-7-153 24636154 PMC3985592

[45] Mutombo N., et al, Benefits of family planning: an assessment of women’s knowledge in rural Western Kenya. BMJ Open, 2014 4(3): p. e004643 10.1136/bmjopen-2013-004643 24643170 PMC3963367

[46] Garrett C.C., et al, Understanding the low uptake of long-acting reversible contraception by young women in Australia: a qualitative study. BMC Womens Health, 2015 15(1): p. 015–0227. 10.1186/s12905-015-0227-9 PMC4566517 26359250

[47] Anguzu R., et al, Knowledge and attitudes towards use of long acting reversible contraceptives among women of reproductive age in Lubaga division, Kampala district, Uganda. BMC Res Notes, 2014. 10.1186/1756-0500-7-153 PMC3985592 24636154

[48] Credé S., et al, Factors impacting knowledge and use of long acting and permanent contraceptive methods by postpartum HIV positive and negative women in Cape Town, South Africa: a cross-sectional study. BMC Public Health, 2012 12(197): p. 1–9. 22424141 10.1186/1471-2458-12-197 PMC3328250

[49] Mekonnen G., et al, Prevalence and factors affecting use of long acting and permanent contraceptive methods in Jinka town, Southern Ethiopia: a cross sectional study. Pan Afr Med J, 2014 18: p. 98 10.11604/pamj.2014.18.98.3421 25404960 PMC4232023

[50] Bulto G.A., Zewdie T.A., and Beyen T.K., Demand for long acting and permanent contraceptive methods and associated factors among married women of reproductive age group in Debre Markos Town, North West Ethiopia. BMC Womens Health, 2014 14(1): p. 46 10.1186/1472-6874-14-46 24625360 PMC3975156

[51] Ochako R., et al, Barriers to modern contraceptive methods uptake among young women in Kenya: a qualitative study. BMC Public Health, 2015 15(118): p. 1–9. 25884675 10.1186/s12889-015-1483-1 PMC4336491

[52] Mekonnen G., et al, Prevalence and factors affecting use of long acting and permanent contraceptive methods in Jinka town, Southern Ethiopia: a cross sectional study. Pan Afr Med J, 2014 18(98): p. 1–8. 10.11604/pamj.2014.18.98.3421 PMC4232023 25404960

[53] Anguzu R., et al, Knowledge and attitudes towards use of long acting reversible contraceptives among women of reproductive age in Lubaga division, Kampala district, Uganda. BMC Res Notes, 2014 7(153): p. 1–9. 24636154 10.1186/1756-0500-7-153 PMC3985592

[54] Moreau C., et al, IUD use in France: women’s and physician’s perspectives. Contraception, 2014 89(1): p. 9–16. 10.1016/j.contraception.2013.10.003 24239330

[55] Meskele M. and Mekonnen W., Factors affecting women’s intention to use long acting and permanent contraceptive methods in Wolaita Zone, Southern Ethiopia: A cross-sectional study. BMC Womens Health, 2014 14(109): p. 1–9. 25216640 10.1186/1472-6874-14-109 PMC4237819

[56] Peipert J.F., et al, Continuation and Satisfaction of Reversible Contraception. Obstet Gynecol., 2011 117(5): p. 1105–1113. 10.1097/AOG.0b013e31821188ad 21508749 PMC3548669

[57] Greenberg K.B., Makino K.K., and Coles M.S., Factors associated with provision of long-acting reversible contraception among adolescent health care providers. J Adolesc Health, 2013 52(3): p. 372–4. 10.1016/j.jadohealth.2012.11.003 23427785 PMC3725589

[58] Tekelab T., Sufa A., and Wirtu D., Factors Affecting Intention to Use Long Acting and Permanent Contraceptive Methods among Married Women of Reproductive Age Groups in Western Ethiopia: A Community Based Cross Sectional Study. Family Medicine & Medical Science Research, 2015 4(1): p. 1–5.

[59] White K., et al, Knowledge and Attitudes about Long-Acting Reversible Contraception Among Latina Women Who Desire Sterilization. Women’s Health Issues 23–4, 2013 23(7): p. e257–e263. 10.1016/j.whi.2013.05.001 PMC3707629 23816156

[60] Katz K.R., et al, Reasons for the Low Level of IUD Use in El Salvador. International Family Planning Perspectives, 2002 28(1): p. 26–31.

[61] Asker C., et al, What is it about intrauterine devices that women find unacceptable? Factors that make women non-users: a qualitative study. BMJ Sexual and Reprod Health, 2006 32(2): p. 89–94. 10.1783/147118906776276170 16824298

[62] Dhont N., et al, Improved access increases postpartum uptake of contraceptive implants among HIV-positive women in Rwanda. Eur J Contracept Reprod Health Care, 2009 14(6): p. 420–5. 10.3109/13625180903340584 19929645

[63] Mota K., Reddy S., and Getachew B., Unmet need of long-acting and permanent family planning methods among women in the reproductive age group in shashemene town, Oromia region, Ethiopia: a cross sectional study. BMC Womens Health, 2015 15(51): p. 1–8. 26174238 10.1186/s12905-015-0209-y PMC4502950

[64] Kohan S., Talebian F., and Ehsanpour S., Association between women’s autonomy and family planning outcome in couples residing in Isfahan. Iran J Nurs Midwifery Res, 2014 19(5): p. 451–5. 25400671 PMC4223960

[65] Fekadu H, K. A., Yesuf EA, Hussien G, Tafa M., Prevalence and Determinant Factors of Long Acting Contraceptive Utilization among Married Women of Reproductive Age in Adaba Town, West Arsi Zone, Oromia, Ethiopia. Journal of Women’s Health Care, 2017 6(1): p. 1–11.

