# Supplementary material for: Determinants of long acting reversible contraception utilization in Northwest Ethiopia: An institution-based case control study
Source: PLoS One. 2020 Oct 20;15(10):e0240816. doi: 10.1371/journal.pone.0240816 (PMC7575092; doi:10.1371/journal.pone.0240816)
Supplement: S1 Tool — (ZIP) [file pone.0240816.s001.zip › Amharic tool-converted.pdf]

በጎንደር ዩኒቨርሲቲ ህክምናና ጤና ሳይንስ ኮሌጅ

በአማራ ክልል በቤተሰብ ዕቅድ ተጠቃሚ እናቶች ላይ የዘመናዊ የረጅም ጊዜ የወሊድ መከላከያን ከመጠቀም ጋር ወሳኝነት ያላቸውን ዋና ዋና ምክንያቶች ለማጥናት የተዘጋጀ መጠይቅ ነው።

መግቢያ

እኔ-----------እባላለሁ ጎንደር ዩኒቨርሲቲ በሚሰራው የዚህ ጥናት አባል ነኝ፤ ይህ ጥናት የወሊድ መከላከያን በሚጠቀሙ እናቶች ላይ የረጅም ጊዜ የወሊድ መከላከያን ከመጠቀም ጋር ወሳኝነት ያላቸውን ዋና ዋና ምክንያቶች ለማጥናት የተዘጋጀ ነው። ከዚህ በመቀጠል የተወሰኑ ጥያቄዎች እጠይቅዎታለሁ፣ የዚህ ጥናት ውጤት የረጅም ጊዜ የወሊድ መከላከያን ተጠቃሚነት ለመጨመር አዲስ የመፍትሄ መንገዶችን ለማምጣት ይረዳል ። ለዚህ ጥናት ስኬታማነት የሚሰጡት ማንኛውም መረጃ ሚስጥራዊነቱ የተጠበቀና ስሞዎትም የማይፃፍ ከመሆኑም ባሻገር መልስ የሰጡበትን ወረቀት የራሱ ኮድ ተሰጥቶትና ተቆልፎ የሚቀመጥ ነው ። በዚህ ጥናት አለመሳተፍ ወይም ላአንዳንድ ጥያቄዎች መልስ አለመስጠት እና በፈለጉት ሰዓት ማቋረጥ ይቻላል። እርስዎ የሚሰጡን ትክከለኛ መልስ የዘመናዊ የረጅም ጊዜ የወሊድ መከላከያን ከመጠቀም ጋር ወሳኝነት ያላቸውን ዋና ዋና ምክንያቶች ለማጥናት እና ወደፊት ለቤተሰብ ምጣኔ አገልግሎት መሻሻል ትልቅ አስተዋጽኦ ያበረክታል። የምናደርግልዎ ቃለ መጠይቅ ከሰላሳ ደቂቃ የማይበልጥ ሲሆን ለሚያደርጉልን ትብብር በቅድሚያ እናመሰግናለን።

001. በምርምሩ ለመሳተፍ ፈቃደኛ ነዎት?

1. አዎ
2. የለም

002.ክፍለ ከተማ ___________________________

ጤና ተቋም___________________

003. የመረጃ ሰብሳቢው ፊርማ ....

ስም_____________________________

ፊርማ ______________________________

ቀን__________________

ውጤት

1.ሙሉ በሙሉ ተሞልቷል 2. ተቀግዷል 3. በከፊል ተመልሷል

የመጠይቅ መለያ ቁጥር:------------------------   የጤና ተቋም ቁጥር. -------------------------------

ጥያቄ ቁጥር	*ጥያቄ*	*ምርጫ*	*ዝለል*
ክፍል አንድ: **ማህበራዊና ስነ- ህዝባዊ ገጽታች**			
101	እድሜ	.... ዓመት	
101	ሐይማኖት	1. ኦርቶዶክስ<br>2. ሙስሊም<br>3. ካቶሊክ<br>4. ፕሮቲስታንት<br>99. ሌላ ካለ(ይጠቀስ)-------------	
102	የመኖሪያ ቦታ	1. ከተማ<br>2. ገጠር	
103	ብሄር	1. አማራ<br>2. ትግሬ<br>3. ኦሮሞ<br>99. ሌላ ካለ ይገለፅ-------	
104	የጋብቻ ሁኔታ	1. ያገባች<br>2. ያላገባች<br>3. የፈታች<br>4. የሞተባት<br>5. የተለያየች	
105	የት/ደረጃ	1. መደበኛ ት/ት ያልተማረ<br>2. እየተማሩ ከሆነ--------ክፍል<br>3. ዲፕሎማ (10+4 ወይም 12+2)<br>4. የመጀመሪያ ዲግሪና ከዛ በላይ	
106	የባለቤትዎ የት/ት ደረጃ	1. መደበኛ ት/ት ያልተማረ<br>2. እየተማሩ ከሆነ--------ክፍል<br>3. ዲፕሎማ (10+4 ወይም 12+2)<br>4. የመጀመሪያ ዲግሪና ከዛ በላይ	
107	የስራ ሁኔታ	1. የቤት እመቤት<br>2. የመንግስት ቅጥር<br>3. በግል<br>4. የቀን ሰራተኛ	

		5. ገበሬ<br>6. ተማሪ<br>99. ሌላ ካለ(ይጠቀስ)--------------	
108	የባለቤትዎ የስራ ሁኔታ	1. የመንግስት ቅጥር<br>2. የግል ቅጥር<br>3. የቀን ሰራተኛ<br>4. ገበሬ<br>5. ተማሪ<br>99. ሌላ ካለ(ይጠቀስ)--------------	
109	የቤተሰብዎ ወርሃዊ ገቢ(በብር)	-----------ብር	

ጥያቄ ቁጥር	*ጥያቄ*	*ምርጫ*	*ዝለል*
ክፍል ሁለት፡ የስነ ተዋልዶ ታሪክንና የጤና ሁኔታን የተመለከተ ጥያቄ			
201.	ባለትዳር ከነበሩ/ከሆኑ በመጀመሪያ ጋብቻዎ አድሜዎ ስንት ነበር	……. ዓመት	
202.	የእርግዝና ቅጥር(የተፈለገና ያለተፈለገ)	____________የተፈለገ<br>_____________ያለተፈለገ	*መልሱ "0" ከሆነ ወደ 2021*
203.	ስንት ጊዜ ወልደዋል	________	
204.	በህይወት የተወለዱ ስንት ልጆች ናቸው	________	
205.	አስወርዶዎት ያውቃል (በድንገት ሳይታሰብ እና ታስቦበት)	_________በድንገት ሳይታሰብ<br>_____________ታስቦበት	
206.	ስንት ጊዜ ጽንስ ከሆድሽ ውስጥ ሞቶ/ጠፍቶ ተወልዷል	_________	
207.	ስንት ጊዜ አንድ ዓመት ሳይሞላው ልጅ ሞቶብሽ ያውቃል	________	

208.	የመጀመርያ ልጅሽን ስትወልጅ እድሜሽ ስንት ነበር?	_________ዓመት	
209.	በቅርብ በወለድሽው/ሻት እና ከሱ/ከሷ በፊት በወለድሽው/ሻት ልጆችሽ መካከል ልዩነታቸው የምን ያህለ ጊዜ ነው?	1. የመጀመሪያየ ነው<br>2. ______ወራት	
2010.	በመጨረሻ ለወለዱት ልጅ የእርግዝና ክትትል ነበርዎ?	1.አዎ<br>2. የለም	የለም-- ወደ 2012
2011.	አዎ ከሆነ መልስዎ፣ በክትትልዎ ወቅት ስንት ጊዞ ጉብኝት ነበርዎ?	________ ጊዜ	
2012.	በመጨረሻ ልጅዎን መቸ ነበር የወለዱት?	ቀን___ ወር____ ዓ/ም____	
2013.	በመጨረሻ ልጅዎን የት ነበር የወለዱት?	1.ጤና ተቋም<br>2. ቤት<br>ሌላ ካለ (ይጠቀስ) ________________________	
2014.	በቅርብ የነበረው እርግዝናዎ የተፈለገ ነበር	1. አዎ<br>2. የለም	
2015.	መልስዎ የለም ከሆነ፣ ሳይፈልጉ ያረገዙበት ምክንያትዎ ምን ነበር	1. የወሊድ መከላከያ ስለማልጠቀም<br>2. የወሊድ መከላከያ ያለመሳካት/ውድቀት<br>99. ሌላ ካለ ይጠቀስ-----	
2016.	ምክንያትዎ የወሊድ መከላከያ ያለመሳካት/ውድቀት ከሆነ፣ የሚጠቀሙት የመከላከያ ዘዴ ምን ነበር	------------------------	
2017.	በቅርብ ለወለዱት ልጅዎ የድኅረ ወሊድ ክትትል ነበርዎ	1. አዎ<br>2. የለም	
2018.	በቅድመ ወሊድ ክትትልዎ ጊዜ የቤተሰብ ምጣኔ የምክር አገልግሎት አግኝተው ነበር	1. አዎ<br>2. የለም	
2019.	በድኅረ ወሊድ ክትትልዎ ጊዜ የቤተሰብ ምጣኔ የምክር አገልግሎት አግኝተው ነበር	1. አዎ<br>2. የለም	

2020.	በቅርብ ለወለዱት ልጅዎ ጡት አጥብተውታል	1. አዎ<br>2. የለም	
2021.	ስንት ልጆች እንዲኖሩሽ ትፈልጊአለሽ	1.______ወንዶች<br>2.______ሴቶች	
2022.	በአሁኑ ወቅት የመውለድ ሃሳብሽ ወይም ግብሽ ምንድን ነው	1. ማራራቅ እፈልጋሉ<br>2. ማቆም እፈልጋለሁ<br>3. አልወሰንኩም<br>4. በቅርቡ መውለድ እፈልጋለሁ	
2023.	ሲጋራ ያጨሳሉ	1. አዎ<br>2. የለም	
2024.	ስር የተደደ/የቆየ በሽታ አለብዎ	1. የለብኝም<br>2. ኤችአይቪ<br>3. ስኳር<br>4. የደም ግፊት<br>5. የልብ ድካም<br>6. የኩላሊት መድከም<br>7. ሌላ(ይጠቀስ)--------	

ጥያቄ ቁጥር	ጥያቄ	ምርጫ	ዝለል
ክፍል 3፡ የወሊድ መከላከያ አጠቃቀምን እና የረዥም ጊዜ የመሊድ መከላከያ እውቀትን የተመለከተ ጥያቄ			
301.	ስለ ረጅም ጊዜ የመሊድ መከላከያ መንገዶች ሰምተው ያውቃሉ?	1.አዎን<br>2.የለም	የለም ከሆነ ወደ ጥ-304
302.	ከየትኛው የመረጃ ምንጭ ነበር የሰሙት?	1. ሬዲዮ<br>2. ቴሌቪዥን<br>3. መጽሄት/ጋዜጣ<br>4. ከመደበኛ ት/ት<br>5. ከጤና ባለሞያ<br>6. ሌላ (ይጠቀስ)________	
303.	የረጅም ጊዜ ወሊድ መከላከያ የምንላቸው እነማን	1. ክንድ ላይ የሚቀበር<br>2. በማኅጸን የሚቀመጥ	

	ናቸው?		
304.	ከአሁን በፊት ማናቸውንም ዘመናዊ የወሊድ መከላከያ ዘዴዎች ተጠቅመው ያውቃሉ?	*1.አዎን*<br><br>*2.የለም*	*የለም ከሆነ ወደ ጥ-308*
305.	አዎ ከሆነ መልስዎ፣ የትኛውን ዓይነት የወሊድ መከላከያ ዘዴ ነበር የሚጠቀሙ? (ከአንድ በላይ መመለስ ይቻላል)	1.መርፌ<br><br>2. ታብሌት<br><br>3. ከክንድ የሚቀበር<br><br>4. በማኅጸን የሚቀመጥ<br><br>5.የወነድ ኮንዶም<br><br>6. የሴት ማምከን<br><br>7. የወንድ ማምከን<br><br>*99. ሌላ (ይጠቀስ)________*	
306.	የሚጠቀሙትን የወሊድ መከላከያ ዘዴ የት ነው የሚያገኙት	1. ሆስፒታል<br><br>2. ጤና ጣቢያ<br><br>3.ጠየና ኬላ<br><br>4. ከግል የጤና ተቋም<br><br>*99. ሌላ (ይጠቀስ)________*	
307.	በሚጠቀሙት የወሊድ መከላከያ ዘዴ አጋጥሞዎ የሚያውቅ የጤና ችግር አለ?	*1.አዎን*<br><br>*2.የለም*<br>88. አላስታውስም	
308.	መልስዎ አዎ ከሆነ፣ከሚከተሉት የትኞቹ የጤና እክሎች ነበር ያጋጠምዎት? ((ከአንድ በላይ መመለስ ይቻላል))	*1, በማኅጸን ጊዜውን ያልጠበቀ የደም መፍሰስ*<br><br>*2,የክብደት መጨመር*<br><br>*3, ማቅለሽለሽና ማስታወክ*<br><br>*4, የሆድ ህመም*<br><br>*5, በሚቀበርበት ቦታ የሚከሰት ቁስለት*	

		6, ሌላ ካለ(ይጠቀስ)________	
309.	በአሁኑ ሰዓት ምን ዓይነት የወሊድ መከላከያ ዘዴ ነው የሚጠቀሙ?	1.መርፌ<br>2. ታብሌት<br>3. ከክንድ የሚቀበር<br>4. በማኅጸን የሚቀመጥ<br>5.የወነድ ኮንዶም<br>6. የሴት ማምከን<br>7. የወንድ ማምከን<br>*99. ሌላ (ይጠቀስ)________*	
3010.	በአሁኑ ሰዓት የሚጠቀሙትን የወሊድ መከላከያ ዘዴ ለምን መረጡት?	1. በጣም ውጤታማ ስለሆነ<br>2. ተስማሚ/ምቹ ስለሆነ<br>3. ሳቆም መውለድ ስለምችል<br>4. የጎንዮሽ ችግሩ አናሳ ስለሆነ<br>5. በቀላሉ ስለሚገኝ<br>6. ለረዥም ጊዜ ስለሚወሰድ<br>7. ምልልሱ አነስተኛ ስለሆነ<br>8.ምንም አላስታውስም<br>*99. ሌላ (ይጠቀስ)________*	
3011.	የምትጠቀሚውን የወሊድ መከላከያ የሚያቀርብልሽ ማን ነው?	1. የማኅጸንና ጽንስ ስፔሻሊስት<br>2. ጠቅላላ ሀኪም<br>3. አዋላጅ ነርስ<br>4. ነርስ<br>5. የጤና መኮንነ<br>*99. ሌላ (ይጠቀስ)________*	
3012.	አሁን የወሊድ መከላከያ ዘዴውን ያቀረበልሽን የጤና ባለሞያ ምክር ታምኝዋለሽ?	1. አዎ<br>2.የለም<br>3. አስተያየት የለኝም	
3013.	የሚጠቀሙት የአጭር ጊዜ የወሊድ መከላከያ ዘዴ ከሆነ በምን ምክልያት ነው የረጅም ጊዜ የወሊድ	1. የጎንዮሽ ችግር ስለምፈራ<br>2. እንዳያመክነኝ ስለምፈራ	የረጅም ጊዜ የወሊድ

	መከላከያ የማይጠቀሙ? (ከአንድ በላይ መመለስ ይቻላል)	3. ብዙ ልጆች እንዲኖሩኝ ስለምፈልግ<br>4. ኃይማኖቴ ስለሚከለክል<br>5. የግል የጤና ችግር ስላለብኝ<br>6. የምፈልገው ዘዴ ስለሌለ<br>7. አሉባልታ<br>8. ባህላችን ስለማይፈቅድ<br>9. ጠቃሚ በሆኑ በሌሎች ተጽእኖ<br>10.በእውቀት ማነስ<br>11. መርፌና ህመም ስለምፈራ<br>12. የገቢ ምንጭ ስለሚያንሰኝ<br>*99. ሌላ (ይጠቀስ)*_______	መከላከያ የሚጠቀሙ ከሆነ ወደ---
3014.	የሚጠቀሙትን የወሊድ መከላከያ ዘዴ ለምን ዓላማ ነው የሚጠቀሙት?	1. ለማራራቅ<br>2.ለመመጠን<br>*99. ሌላ (ይጠቀስ)*_______	
3015.	የመረጡትን የመከላከያ ዘዴ ነው እየተጠቀሙ ያሉ?	0.መልስ የለኝም<br>1.አዎን<br>2.የለም	
3016.	መልስዎ የለም ከሆነ፣ ምክንያቱ ምንድን ነው?	1. የመከላከያ ዘዴ አለመኖር<br>2. የጤና ባለሞያ ተጽእኖ<br>3. የባለቤቴ ምርጫ<br>4. ሌላ(ይጠቀስ)____________	
3017.	የሚጠቀሙት የረጅም ጊዜ የወሊድ መከላከያ ዘዴ ከሆነ በምን ምክልያት ነው የአጭር ጊዜ የወሊድ መከላከያ የማይጠቀሙ? (ከአንድ በላይ መመለስ ይቻላል)	1. የጎንዮሽ ችግር ስለምፈራ<br>2. እንዳይከሽፍብኝ ስለምፈራ<br>3. ብዙ ልጆች እንዲኖሩኝ ስለማልፈልግ<br>4. የግል የጤና ችግር ስላለብኝ<br>6. የምፈልገው ዘዴ ስለሌለ<br>7. አሉባልታ<br>8. ባህላችን ስለማይፈቅድ<br>9. ጠቃሚ በሆኑ በሌሎች ተጽእኖ<br>10. ለማስታወስ ስለምቸገር<br>11. በባለቤቴ ተጽእኖ<br>*99. ሌላ (ይጠቀስ)*______	*If you are SARC user go to 319*
3018.	በቅርብ የወለዱት (ትንሹ ልጅዎ) እድሜው ስንት ሲሆን ነበር የመሊድ መከላከያ መጠቀም የጀመሩ?	__________ሳምንታ ________ወራት	

3019.	ባለቤትዎ ስለወሊድ መከላከያ ዘዴ ጥሩ እውቀት ብለው ያስባሉ?	1. አዎ<br><br>2. የለም	
3020.	ባለቤትዎ ስለ ወሊድ መከላከያ ዘዴ መልካም አመለካከት ያለው ይመስልዎታል	1. አዎ<br><br>2. የለም	
3021.	አሁን እየተጠቀሙት ያለውን የወሊድ መከላከያ ዘዴ እንዲጠቀሙ የወሰነው ማነው?	*1.እኔ ራሴ*<br><br>*2.በዋነኝነት ባለቤቴ*<br><br>*3.ከባለቤቴ ጋር በጋራ*<br><br>*4.የጤና ባለሞያ*<br><br>*99.ሌላ(ይጠቀስ)*__________	
3022.	አሁን እየተጠቀሙት ስላለው የወሊድ መከላከያ ዘዴ ከባለቤትዎ ጋር ተወያይተው ያውቃሉ? ?	1. አዎ<br><br>2. የለም	
3023.	የወሊድ መከላከያ ግልጋሎትና ተያያዥ ጉዳዮችን በተመለከተ የባለቤትዎትን ውሳኔ ይፈልጋሉ(ለምሳሌ፡ የገንዘብ ድጋፍ፣ቀጠሮ ማስታወስ፣መከላከያ ዘዴዎችን በመምረጥ መሳተፍ)?	1. አዎ<br><br>2. የለም	

**4. ስለረዥም ጊዜ የወሊድ መከላከያ ዘዴ አመለካከት የተመለከተ መጠይቅ**							
		በጣም እስማማለሁ	አልስማማ ም	እርግጠኛ አይደለሁ ም	እስማ ማለሁ	በጣም እስማማለሁ	
401.	**የረዥም ጊዜ የወሊድ መከላከያ ዘዴ መጠቀም ለእኔ**	1<br><br>እጅግ በጣም ከባድ ነው	2<br><br>ከባድ ነው	3<br><br>እርግጠኛ አይደለሁም	4<br><br>ቀላል ነው		
402.	**የረዥም ጊዜ የወሊድ መከላከያ ዘዴ መጠቀም**	1<br><br>ፈጽሞ	2	3<br><br>እርግጠኛ	4		

		አይቻልም	አይቻልም	አይደለሁም	ይቻላል		
403.	**የረዥም ጊዜ የወሊድ መከላከያ ዘዴ መጠቀም ለእኔ**	1<br>እጅግ በጣም መጥፎ ነው	2<br>መጥፎ ነው	3<br>እርግጠኛ አይደለሁም	4<br>ጥሩ ነው		
404.	**የረዥም ጊዜ የወሊድ መከላከያ ዘዴ መጠቀም**	1<br>እጅግ በጣም ዋጋ ቢስ ነው	2<br>ዋጋ ቢስ ነው	3<br>እርግጠኛ አይደለሁም	4<br>ጠቃሚ ነው		
405.	**የረዥም ጊዜ የወሊድ መከላከያ ዘዴ መጠቀም**	1<br>እጅግ በጣም ደስ አይልም	2<br>ደስ አይልም	3<br>እርግጠኛ አይደለሁም	4<br>ደስ ይላል		
406.	**የረዥም ጊዜ የወሊድ መከላከያ ዘዴ መጠቀም** ---	1<br>በጣም ጎጅ ነው	2<br>ጎጅ ነው	3<br>እርግጠኛ አይደለሁም	4<br>ጠቃሚ ነው		

5፣. የተሳሳቱ አመለካከቶች

501)		በጣም እስማማለሁ	አልስማማም	እርግጠኛ አይደለሁም	እስማ ማለሁ	በጣም እስማ ማለሁ	
501)	የረጅም ጊዜ የወሊድ መከላከያ ዘዴ ያልተፈለገ እርግዝናን እንድከላከል ይረዳኛል	1	2	3	4	5	
502)	የረጅም ጊዜ የወሊድ መከላከያ ዘዴ መጠቀም ከእርግዝናና ወሊድ ጋር ከተያያዙ ችግሮች ነጻ እንድሆን ይረዳኛል	1	2	3	4	5	
503)	የረጅም ጊዜ የወሊድ መከላከያ ዘዴ መጠቀም	1	2	3	4	5	

	በየቀኑ ታብሌት ከመርሳት ይጠብቀኛል						
504)	የረጅም ጊዜ የወሊድ መከላከያ ዘዴ መጠቀም የጎንዮሽ ችግር ያመጣብኛል	1	2	3	4	5	
505).	የረጅም ጊዜ የወሊድ መከላከያ ዘዴ መጠቀም በስራየ ውጤታማ እንድሆን ይረዳኛል	1	2	3	4	5	
506).	የረጅም ጊዜ የወሊድ መከላከያ ዘዴ መጠቀም ከባለቤቴ ጋር አርኪ የሆነ ግንኙነት እንዲኖረኝ ይረዳኛል	1	2	3	4	5	
507	የረጅም ጊዜ የወሊድ መከላከያ ዘዴ መጠቀም ስለ መከላከያ ዘዴው ጥቅም መረጃ እንዳገኝ ይረዳኛል	1	2	3	4	5	
508	የረጅም ጊዜ የወሊድ መከላከያ ዘዴ መጠቀም ለችግር ያጋልጠኛል	1	2	3	4	5	
509	የረጅም ጊዜ የወሊድ መከላከያ ዘዴ መጠቀም ጊዜና ገንዘብ ይቆጥብልኛል	1	2	3	4	5	
510	የረጅም ጊዜ የወሊድ መከላከያ ዘዴ መጠቀም ሴቶች ላይ መካንነት ያመጣል	1	2	3	4	5	
