# Supplementary material for: Determinants of long acting reversible contraception utilization in Northwest Ethiopia: An institution-based case control study
Source: PLoS One. 2020 Oct 20;15(10):e0240816. doi: 10.1371/journal.pone.0240816 (PMC7575092; doi:10.1371/journal.pone.0240816)
Supplement: S1 Tool — (ZIP) [file pone.0240816.s001.zip › EnglishTool-converted.pdf]

University of Gondar, college of Medicine and Health Sciences

A Questionnaire prepared for assessing factors influencing LARC utilization among modern contraceptive user in the reproductive age group.

Participant information sheet and consent form

Title: Determinants of long acting reversible contraception utilization among modern contraceptive users in North West Ethiopia: institution based case control study

Invitation to participate in the study

Good morning/afternoon, we invite you to participate in a research study. This information leaflet will give you information to help you decide if you want to participate. Before you agree to this, you should fully understand what is involved before you agree to participate in this study. If you decide to be part of this research, we will ask you to sign and date your agreement in an informed consent form at the end of this document. If you have any questions that this leaflet does not fully explain, please do not hesitate to ask the researcher.

Who is doing this research?

This research is being conducted by the research teams at University of Gondar, Department of Gynecology and Obstetrics and Institute of Public Health.

What is the nature and purpose of this study?

The aim of this study is to assess KAP on PP FP among provider and client perspectives before and after the intervention in public health facility in Gondar city. We will interview postpartum women between the ages of 18 to 49. We are asking you to participate in this study because you fall in this age group. If you agree to participate in this study, you will be one of approximately 662 participants. As a participant in this study, you are likely to be interviewed once by a trained female or male researcher who will ask you about your background, socio-demographic and economic characteristics of family planning user in a public health facility, reproductive history and utilization of family planning. The interview will last about 40 minutes. No one except the researcher will know what information you have given in the study.

Possible Risks and Discomforts

You might not be comfortable with some questions in the questionnaire. If that happens, please feel free not to answer questions that you are not comfortable with. If you choose not to answer questions you are not comfortable with or withdraw from the study, this will not affect you in any way and will not affect your relationship with the researchers. If during the interview you find information that disturbs you emotionally, please inform the researcher.

Are there any Costs involved for me?

Apart from the time you may spend completing the questionnaire there are no costs to you in participating in this study.

Possible Benefits

There are no direct benefits to you in participating in this study. But, your participation in this study will provide useful information that may help in the future implementation of interventions to increase postpartum family planning use in Ethiopia.

Confidentiality

All information received from you in this interview will be kept in strict confidence and used for research purposes only. All hard copies of data (information) including study material will be stored in locked cabinets at the project office located at the department of Gynecology, University of Gondar. All soft copies (electronic version) of data will be stored unto password projected computers in the department of Gynecology, University of Gondar. All data will be kept in secure storage for 5 years, thereafter it will be destroyed. Participants will not be identified by name during data entry, and therefore we will collect your information in an anonymous way, that is, without your identity. In this way we will ensure no one else apart from the research will know what information you have shared in the interview. Findings of this study will be published in professional journals in a way that will not be possible to identify you as a participant in this study.

Reimbursement

You will be reimbursed for the time you spend and the inconvenience you may have had in completing the questionnaire. This money will be given to you at the end of this interview as a show of our appreciation for your participation in this study.

What are my rights as a participant?

Your participation in this study is completely voluntary. You can chose not to participate in this study at all, and this will not affect you in anyway. Also, you may agree to participate in this study and later decide to withdraw from the study at any point and there will be no negative consequences to you.

Contacts for Additional Information

In case you have further questions regarding this study, please direct them to **Dr Kiros Terefe** on telephone number **+251 911784112**

Has the study received ethical approval?

This protocol has been structured in accordance with the ethical guidelines and principles of the Declaration of Helsinki (2013). This research study has been approved by the University of Gondar Ethical Review Committee (IRB). These ethics committees will continue to see if this study is being done in a safe way until the study is completed. I have copies of approval letters from the ethics committees that show that the study was approved by them. A copy can be given to you to if you wish to have one.

If you have any questions about your rights as a research participant you can contact the IRB Office between the hours of 8am-5pm through the landline **+251 58 812 9005**

Questionnaire ID:------------------------ Facility No. -------------------------------

Q.No.	Question	Choices	skip
Part one : **Socio- demographic characteristics**			
101	How old are you?(age in years)	___________year	
102	What is your religion?	1. Orthodox<br>2. Muslim<br>3. Catholic<br>4.Protestant<br>99.Others/specify___________	
103	Residence	1. Urban<br>2. Rural	
104	What is your ethnicity	1. Amhara<br>2. Tigray<br>3.Oromo<br>99.Others/specify _____________________	

105	What is your current marital status?	1. Married<br>2. Single<br>3. Divorced<br>4. widowed<br>5. Separated	
106	What is your educational level?	1.No formal education<br>2. If School, grade level _______<br>3.Deploma (10+4 or 12+2)<br>4 1st Degree and above	
107	What is your partner's educational level?	1.No formal education<br>2. If School, grade level _______<br>3.Deploma (10+4 or 12+2)<br>4 1st Degree and above	
108	What is your current occupation?	1. House wife<br>2. Government employee<br>3. Private employee<br>4. Daily labourer<br>5. Farmer<br>6. Student<br>99.Other(specify)______________	
109	What is your partner's current occupation?	1. Government employee<br>2. Private employee<br>3. Daily laborers<br>4. Farmer<br>5. Student<br>99.Other (specify)____________	
1010	What is your family monthly income?	____________Ethiopian Birr	

Q.No.	Question	Choices	Skip
**Part two: Information on Reproductive history and health status of the participants**			
201.	If you are married, what was your age at first marriage?	______ years	
202.	Number of pregnancy (wanted vs Unwanted)	____________wanted<br>_____________unwanted	*If "0" go to 2021*

203.	Number of delivery	_______	
204.	Number of live births	_______	
205.	Number of abortions and type (spontaneous vs Induced)	__________spontaneous<br><br>____________induced	
206.	Number of still births	________	
207.	Number of infant deaths	_______	
208.	What was your age when you have your first child?	________years	
209.	What was the time gap between the previous and the recent birth (Birth interval)?	1. It is the first time<br>2. _____Enter in month	
2010.	Did you attend ANC for last child ?	*1.Yes*<br><br>*2.No*	*If No skip to Q2012*
2011.	If your answer for Q2010 is Yes, how many visits did you attend?	_______ enter number of visit	
2012.	When did you deliver your last child?	*Date___month____year____*	
2013.	Where was the place of birth for your recent baby?	*1.Health facility*<br><br>*2. Home*<br><br>*If other please spacifay ________________________*	
2014.	Was the recent pregnancy wanted	*1. Yes*<br>*2. No*	
2015.	If no, What was the reason for your unwanted pregnancy	*1. No contraception utilization*<br>*2. Method failuer*<br>*3. Others (specify)_______*	
2016.	If the reason is method failure, what type of contraception were you using?	*1. _______________*	
2017.	Do you receive PNC follow up for you recent baby?	*1.Yes*<br><br>*2.No*	
2018.	Do you receive FP counseling during prenatal care?	*1.Yes*	

		2.No	
2019.	Do you receive FP counseling during postnatal care?	1. *Yes*<br>2. *No*	
2020.	Have you breastfeed your recent child?	1.Yes<br>2.No	
2021.	How many children do you want to have (fertility desire)?	*1._____Male*<br>*2._____Female*	
2022.	What is your current reproductive intention (goal)?	1.Want to have space<br>2.Want to limit<br>3.Undecided<br>4. Want to have a child soon	
2023.	Do you smoke cigarette?	1. *Yes*<br>2. *No*	
2024.	Do you have any chronic medical co-morbidity?	1. *I don't have*<br>2. *HIV*<br>3. *DM*<br>4. *HTN*<br>5. *CHF*<br>6. *RF*<br>7. *Other(specify)_________*	

Q.No.	Question	Choices	Remark
**Part three: Information on contraceptive utilization and Knowledge of LARC**			
301.	Have you ever heard about long acting reversible family planning methods?	*1.Yes*<br>*2.No*	*If No skip to 304*

302.	From which source you have heard?	1. *Radio*<br>2. *TV*<br>3. *Megazines/news paper*<br>4. *From formal class*<br>5. *From a health professional*<br>6. *Other(specify)_______*	
303.	If Q 301 Yes, which are those methods?	1. Implant<br>2. IUD	
304.	Have you ever used any modern method of contraceptives prior to the current time?	*1.Yes*<br>*2.No*	*If no go to Q 308*
305.	If yes, which contraceptive method(s) were you using? (multiple answers are possible)	1.Injectable<br>2. Pills<br>3. Implants<br>4. IUD<br>5.Male condoms<br>6. Female Sterilization<br>7. Male sterilization<br>99. other specify________	
306.	From where are you getting the Contraceptions	1. Hospital<br>2. health center<br>3.health post<br>4. private health institution<br>5. others(specify)________________	
307.	Did you experience any health problem(s) with the method(s)?	*1.Yes*<br>*2.No*<br>88. Don't remember	
308.	If yes for Q 306, which of the following health complications have you experienced? (multiple answers are possible)	*1, irregular vaginal bleeding*<br>*2,Weight gain*<br>*3, Nausia and vomiting*<br>*4, Abdominal pain*	

		5, insertion site infection<br>*6, if other please specifay_________*	
309.	Which contraceptive method(s) are you currently using?	1.Injectable<br>2. Pills<br>3. Implants<br>4. IUD<br>5.Male condoms<br>6. Female Sterilization<br>7. Male sterilization<br>99. other specify_____________	
3010.	Why do you prefer the method you are using?	1. Very effective<br>2. It is convenient<br>3. Reversible<br>4. Fewer side effects<br>5. Easley available<br>6. Long acting<br>7. Convenient<br>8. Nothing to remember but the return visit<br>9. Other, specify___________	
3011.	Who is providing you the contraceptive method you are using?	1. An Obstetrician and Gynecologist<br>2. A General practitioner<br>3. A midwife<br>4. A nurse<br>5. A health officer<br>6. Other(specify)_____	
3012.	Do you trust the advise you get from your current provider?	1. Yes<br>2. No<br>3. no opinion	
3013.	If you are using SARC, why do you not use the LARCs? (multiple answers are possible)	1. Fear of side effects<br>2. Fear of infertility<br>3. Desire to have more children<br>4. Religious prohibition<br>5. Medical problem	*If you are LARC user go to*

		6. Preferred method not available<br>7. Rumors<br>8. Unacceptable in my culture<br>9. Important others influence<br>10.Lack of knowledge<br>11. fear of needle and pain<br>12. lack of commodity<br>12.others, specify____________	
3014.	For what purpose are you using the methods?	1.Spacing<br>2.Limiting<br>3. Other(specify)__________	
3015.	Are you using the method of choice?	0.No response<br>1.Yes<br>2.No	
3016.	If no, what is the reason?	1. Unavailability of the method<br>2. Health care provider influence<br>3. My husband preference<br>4. Other(specify)_____________	
3017.	If you are currently using LARCs, What is your reason not to choose SARC? (multiple answers are possible)	1. Fear of side effects<br>2. Fear of method failure<br>3. Desire not to have more children<br>4. Medical problem<br>5. Preferred method not available<br>6. Rumors<br>7. Unacceptable in my culture<br>8. Important others influence<br>9. difficult to recall (not convenient)<br>10. partner influence<br>10. others, specify____________	*If you are SARC user go to 319*
3018.	For what purpose are you using the methods?	1.Spacing<br>2.Limiting<br>3. Other(specify)__________	
3019.	Are you using the method of choice?	0.No response<br>1.Yes<br>2.No	

3020.	If no, what is the reason?	1. Unavailability of the method<br>2. Health care provider influence<br>3. My husband preference<br>4. Other(specify)____________	
3021.	At what age of your recent child did you start using the family planning method?	__________ weeks/_______Months	
3022.	Do you think your partner has good knowledge about contraceptive methods?	1. Yes<br>2. No	
3023.	Do you think that your partner has good attitude towards contraceptive utilization.	1. Yes<br>2. No	
3024.	Who decide for you to use the current family planning methods?	*1.My self*<br>*2.Manily my husband*<br>*3.Jointly decision*<br>*4.Health care provider*<br>*99.Others(specify)*__________	
3025.	Have you discussed with your husband on family planning method you are using currently?	*1.Yes*<br>*2.No*	
3026.	Do you need your husband approval (i.e. financial support, reminded the women about the next appointment dates, participated in the FP methods by procuring some contraceptive method) in issues related to family planning services?	*1.Yes*<br>*2.No*	

**4. Attitude about long acting reversible contraceptive methods**							
		Very unlikely	unlikely	uncertain	Likely	Very likely	
401.	For me to use LARCs is	1	2	3	4		

		Extremely difficult	difficult	Not sure	Easy		
402.	To use LARCs is	1<br>Definitely impossible	2<br>Impossible	3<br>Not sure	4<br>Possibl e		
403.	For me to use LARCs is	1<br>Extremely bad	2<br>bad	3<br>Not sure	4<br>Good		
404.	To use LARCs is	1<br>Extremely worthless	2<br>worthless	3<br>Not sure	4<br>Valuab le		
405.	To use LARCs is	1<br>Extremely unpleasant	2<br>Unpleasan t	3<br>Not sure	4<br>Pleasa nt		
406.	To use LARCs is<br>---	1<br>Very harmful	2<br>harmful	3<br>Not sure	4<br>Benefi cial		

1) Myths, misconceptions

501)		Very unlikely	unlikely	uncerta in	likely	Very likely	
501)	Using LARCs<br>will help me to prevent<br>unwanted pregnancy	1	2	3	4	5	
502)	Using LARCs will help me be free from pregnancy and birth related problems	1	2	3	4	5	
503)	Using LARCs will save me from forgetting to take<br>daily pills	1	2	3	4	5	

504)	Using LARCs will cause<br><br>me side effects	1	2	3	4	5	
505).	Using LARCs will help me to work effectively	1	2	3	4	5	
506).	Using LARCs will help me to have satisfying<br><br>relationship with my<br><br>husband	1	2	3	4	5	
507	Using LARCs will help me get information about the benefits of it	1	2	3	4	5	
508	Using LARCs will subject<br><br>me to problems	1	2	3	4	5	
509	Using LARCs will help me save time and money	1	2	3	4	5	
510	LARC use causes infertility in a women	1	2	3	4	5	
